# Of the Intentions of Cure in Hydrocephalus

**Published:** 1799-11

**Authors:** 


### *Of the Intentions of Cure in Hydrocephalus.*

[ Continued from p. 258—261 of our laſt. ]

THE remedies which have been moſt commonly reſorted to for the cure of this complaint, are, mercury, internally, as calom pp$^{t}$ ; and, externally, by aborption, as, ung$^{t.}$ hydrargy, F.; bliſters applied on the head, bleedings by leeches, and ſcarifications, ſquills, opium, fol. digit, pur, calx zinci; and lately, the fol. doron German, or German leopard's bane.

Of the firſt of theſe remedies, notwithſtanding the reſpectable evidences of Drs. CARMICHAEL SMITH, DOBSON, QUIN, PERCIVAL, LETTSOM, JOHN HUNTER, HAYGARTH, MOSELY, and ARMSTRONG, other phyſicians and ſurgeons of eminence have not been ſo ſucceſsful in the uſe of it. Salivation is not very eaſily raiſed in young children, eſpecially in this diſeaſe.* In regard to veneſection, either local or general, it is much to be regretted, that neither from the teſtimonies of authors, nor from any obſervations hitherto made, are we led to determine on its uſe. From Dr. QUIN's view of the complaint, we ſhould be led to aſcribe more to inflammation, than what appears to *me* to be the real ſtate of the diſeaſe, and, conſequently, ought to make us very cautious in the uſe of blood-letting, even in the primary ſtage; for the veſſels of the brain ſeem quickly to loſe their tone by diſtenſion, and great atony and torpor of the whole ſyſtem ſucceed. If depletion, therefore, be indicated, it will be beſt accompliſhed by occaſional mercurial purgatives, bliſters in the direction of the ſutures, &c. &c. Practitioners know, that inflammation is often found to exiſt in relaxed habits, excited and kept up by the irritation of ſome peculiar acrimony, totally contrary and independent of any inflammatory diatheſis, as in ſcrophula, lues venerea, &c. &c.

The remedies which I place my chief reliance on, are as follow; arranged in the order in which they will be wanted.

Calomel pp$^{t.}$, to empty the bowels. The form I preſcribe to a child of four years of age is this:

℞ calom pp$^{t.}$ g$^{r.}$ iij, opii g$^{r.}$ ß. cretæ pp$^{t.}$, g$^{r.}$ vi m. f$^{t.}$ pulvis h. s. ſumendus.

Blood-letting.—Leeches applied to the temples, and in a direction

* Dr. MICHAEL LEIB relates the caſe of a child, who took 112 grs. hydrar. muriats mitis in 16 days, with a hydrocephalus.

with with the futures; v. fectio; to be repeated pro re natâ, in the firft ftage of the difeafe.

Bliflers.—Cut in ftrips, about three quarters of an inch broad, and laid over the futures, the head being previoufly fhaved. Alfo to be applied to the head, neck, and between the thighs.

To mitigate the pain and fpafm—
Opium and digitalis combined.

In cafes of ftupor, coma, &c.—

Sal. c. c. vol. fp$^{ts.}$ ætheris vitr. comp. mufk, and camphor.

To promote abforption—

Tinctura cantharid. calx zinci, hydrar. mur. mit.; electricity, flight fhocks paffed in various directions through the head every four or fix hours.

As an univerfal ftimulus—
Oxygen-gas, diluted with atmofpherical air.

A generous diet fhould be enjoined to invigorate the fyftem and alimentary canal in particular.

When the bowels are obftinately coftive, and there is a paucity of urine, I give the following:—

℞ Scillæ pulveris rec$^{t.}$ g$^{r.}$ iij opii purif. g$^{r.}$ ß; calomel pp$^{t.}$ g$^{r.}$ ij M. f. pulvis capiat bis vel. ter die.

Laftly, to ftrengthen the conftitution, and prevent the re-accumulation of fluid, by giving the following medicine:—

℞ decoc$^{t.}$ cinchonæ, ℥ vij; tinc$^{t.}$ cantharid. ʒ ij tinc$^{t.}$ cinch. comp. ℥j M. f$^{t.}$ mixtur capiat cochl. iij larg, ter die; for a child eight years of age.

I have been led to give the tinctura cantharidis in this complaint, from frequent experience of its efficacy in fome cafes of paralyfis, and from reading a paper on its ufe in thefe cafes, by my friend Dr. May, in fome late publication. I have found it an excellent ftimulus. I principally attribute the good effects of this medicine in hydrocephalus internus, to the general ftimulant operation, and to the tendency it has of producing a determination to the fkin; and to augment and diffufe the energy of the brain through the remoter extremities of the body; by joining the bals. Peruv. as below, with it, its virtue feems increafed.

℞ bals.

℞ bals. Peruv. ℈i. vitelli ovi q. s. aq. menth. pip$^{s.}$ ℥vj. ſacch. alb. ℈i. tinc$^{t.}$ canth. g$^{tt.}$ xxiv. M. f$^{t.}$ hauſt, bis die capiendus. Doſis tincturæ cantharidis in præſcripto noviſſimè hauſtu ad gutt. xxxvi augeatur.

The appearances on diſſection are, water collected in greater or leſs quantity, with the fornix in the former caſe raiſed at its anterior extremity, in conſequence of its accumulation; and an immediate opening of communication is thereby formed between the lateral ventricles. From this cauſe too, Dr. BAILEY obſerves,* a part of the water paſſes very readily into the third ventricle, and from thence into the fourth. To aſcertain with any degree of preciſion whether children have had an accumulation of water in the brain, who have been ſuppoſed to have died of hydrocephalus internus, it is neceſſary their heads ſhould be opened immediately after their deaths. It appears to me, that in many of the caſes related by BONNETUS,† ſome deception might have ariſen from a delay in opening the heads of many of his patients who died of an accumulation of water in the brain; for, independent of diſeaſe, I have found that the longer after death diſſection is performed, the greater is the quantity of lymph; and SAUVAGES has noticed the ſame. He thus expreſſes himſelf ſomewhere: Nihil vulgatius quam ſerum in ſinubus cerebri reperiri, ſi longò poſt mortem tempore aperiatur cadaver: quò longius, eò uberius invenietur ſerum.

Such inquiries, however, muſt be purſued under proper reſtrictions, and we ſhould with caution draw our concluſions, when we obſerve parts in a morbid condition, which we had no reaſon to ſuſpect to be diſeaſed from the ſymptoms or complaints which aroſe during the patient's illneſs. Some may, perhaps, be produced in *articulo mortis*; and others may have been the effects of a former diſeaſe, which nature, or ſtrength of conſtitution at that time, happily overcame. Hence we ſhould endeavour minutely to acquaint ourſelves with the riſe and progreſs, effects and ſymptoms of the diſeaſe, during the patient's illneſs, that, by conſidering them with the conſequences produced upon the animal œconomy, we may fix the nature of the diſeaſe upon true and juſt principles. An inattention in this reſpect has frequently confounded and miſtaken effects for cauſes, and aſcribed appearances to complaints they were no ways concerned or connected with.

---

* Morbid Anatomy, p. 440.

† Bonneti Sepulchretum Anatomicum.

